# Unbalanced but Satisfied?

**DOI:** 10.3389/fped.2015.00120

**Published:** 2016-01-11

**Authors:** Asha N. Shenoi

**Affiliations:** ^1^Heinrich A. Werner Division of Pediatric Critical Care Medicine, University of Kentucky, Lexington, KY, USA

**Keywords:** burnout, career, satisfaction, physician, balance

During my fellowship training in critical care, my then 4-year-old daughter drew my picture in shabby scrubs with two dark circles around my eyes, and named it “mommy panda” depicting my sleep deprived and exhausted existence. At that time, I was spending about two and a half hours commuting, braving Atlanta traffic, while my husband pursued his PhD training in New York. My situation as an anchor parent of a young child with no family around and the demands of an emotionally and physically challenging fellowship training generated lots of sympathy and concerns from well-wishers and colleagues. They saw my life as unbalanced, at risk of burnout, and it is true that they had some reasons to worry since the odds were not in my favor. Physicians in general and particularly those exposed to unremitting high level of stress in specialties such as critical care are at high risk of burnout (1), a condition resulting from cumulative stress at work characterized by emotional exhaustion, depersonalization, and lack of personal accomplishment (2, 3). Emotional exhaustion is a key component in physician burnout (2), and the reported incidence in the US ranges from 25 to 60% (4, 5).

To avoid becoming yet another victim, I tried to learn what we already know about the risk and protective factors associated with physician burnout. Available evidence describes a number of work and personal factors often associated with physician burnout. Work characteristics such as heavy workload, poor work organization, conflicts with coworkers, patients and their families, and perceived lack of control of work hours and schedule, specialty choice and practice settings are reported as risk factors in both sexes (6–9). Parenting responsibilities, career and work schedule of a significant other, and the strength of social support are some of the factors identified in studies that have examined life style factors (10). As I understood, rather than individual factors, the interplay between these factors forming tension between personal and professional responsibilities, labeled as “work home interference” appears to be at the heart of burnout (11).

During my training and the first few years of practice, I often found solace in the belief that “things get better with time. Evidence suggests that this may be true as younger physicians have twice the incidence of burn out as older colleagues, and onset may be as early as during training (4). Younger physicians, those like I with <10 years of practice history have the lower career satisfaction and higher rates of both depersonalization and home-work conflicts even though they work fewer hours (12). It seems that if you survive the midcareer, when physicians of both sexes and most specialties are least satisfied and most burned out; you have better odds of being satisfied with work–life balance. However, I do wonder these findings may be due to self-selection or generational difference in attitudes to work and life.

While physician burnout is often a natural and anticipated response to cumulative stress in medicine as a system, I have often wondered, as a woman am I at greater risk of burnout than my male colleagues? How strong is the role of gender in the origin, perceptions and the ways one survive a stressful medical career? These are critically important issues for us to explore as more than half of the graduating physicians in the US are women, though they make only 30% of practicing physicians and 12% of professors in academic medicine (13). One-third of women pediatricians work part time for a better balance between professional life and family life (14). So, when I found studies describing female gender as an independent risk factor for physician burnout (7, 15), I was not surprised. I assumed that this is because women in general have role overload, that they often have a disproportionate share of domestic and or parenting responsibilities and therefore have more work–life conflict. I would later find out that this is not so clear cut as I thought. The nature of the gender-burnout relationship across all occupations remains unclear as only a handful of authors have investigated this relationship directly, and empirical results are mixed. Parental and marital status, often blamed as the major reasons for role overload, did not predict burnout, and some studies have even shown that married women physicians with children have less burnout and are more satisfied compared to their unmarried colleagues (16–18). So the positive association or rather the lack of negative association between marriage and parental status on well-being suggests that work–family conflict may not in and of themselves be the major source of excess burnout among women physicians. While suggesting physicians to get married and have children are not pragmatic solutions to combat distress, nurturing and protecting our personal and professional relationships are keys to avoiding burnout (11).

Another theme that has emerged from studies is the actual or perceived lack of control over work schedule as a significant factor associated with burnout (9, 19). Physicians who felt in control over work hours and schedule have significantly lower burnout even when they worked longer hours (15). Women physicians of all ages perceived significantly less control over their schedule across specialties and practice settings and felt more time pressure (9, 18). This may be partly due to gender-related differences in working style and expectations such as more time and effort to communicate with patients and emphasis on addressing psychosocial and health maintenance issues (18).

Fortunately, I have never felt dissatisfied with my career choice and have always viewed this as an insurance against burnout. Yet, could it be possible to feel genuinely satisfied with your career, but feel exhausted and struggle with symptoms of burnout? Interestingly, career satisfaction is a complex issue and the relationship between career satisfaction and burnout appears bidirectional. Burned out physicians tend to be less satisfied with their career, and physicians who are satisfied were less likely to report high levels of burnout (20). Although studies describe majority of physicians are satisfied with their careers, career satisfaction varies by specialty, income, and age (21, 22). Surprisingly, women are more likely to be satisfied with their career choice compared to men (9). Whether it is due to the different work values and work expectations for women or the phenomenon of paradox of contented female worker, i.e., objectively have poor work quality but report equal or more job satisfaction is not clear.

This also alludes to an inherent problem in interpreting evidence on gender differences in physician burnout and career satisfaction. Women are more open to expressing symptoms of emotional and physical fatigue while men are more likely to shut off and withdraw under stress and depersonalize the experience (23). Thus, it is possible that relationship between gender and burnout may have been exaggerated well beyond the actual size. Women occasionally pay a high price for this conventional assumption while being passed over for challenging assignments and promotions while men suffer from unrecognized burnout. It seems that the origins of burnout and career satisfaction are mostly entrenched in the environment and care delivery system rather than in the gender or personal characteristics of a few susceptible people (24).

Emerging evidence on physician work–life balance challenge some of the conventional beliefs on work–life balance and burnout. Specialties with lowest burnout has shown to score high in work–life balance, yet highest burnout were not necessarily among those least satisfied with work–life balance (24). It has been found that work–life balance is not a predictor of career satisfaction after adjusting for common predictors such as work hours, marital status, children, and control over work (19). In contrast, measures of burnout like emotional resilience and personal accomplishment are strongly associated with career satisfaction, and this relationship is independent of work and demographic factors (19). Hence issues of work–life balance are much less predictive of career satisfaction and physicians even with some amount of imbalanced work–life configuration may remain deeply satisfied with careers.

Nevertheless, I have to admit that the most common sensical advice that I received to prevent burnout is to aim for a work–life balance. As I took this to heart and struggled to achieve a new balance, I became painfully aware of the unevenness in my life. My preoccupation with the need for a work–life balance stressed me, whereas the guilt of missing the mark drove me further into despair, until I started to question my struggle. I realized that in my struggle to achieve a work–life balance, I have been trying to redefine my life by drawing artificial lines between my work, my calling and who I am. I found much help and comfort in my Eastern spiritual roots, to accept my life as it is with a certain level of chaos and unpredictability.

I am blessed with the unique opportunity to make a difference in the lives of children and their families during their most stressful and vulnerable times. These experiences teach me to live consciously in the moment and cherish and enjoy precious moments with my family. In turn, I gain my energy and resilience to continue my work from the love and support of my family. So as I see it, my work and family complement and not compete with each other. I feel fine with reasonable imbalance in my life as I achieve fulfillment from my work and support from my family.

My daughter is now 10 years old and I probed her to see how she perceives balance in my life. She did not quite understood the meaning of balance and asked “*does that mean you are happy leaving for work as well as happy coming home*?” I said “*Yes, something like that*”. Her response was “*well, most days*.” I was satisfied with that answer and on any day will choose being happy over chasing an ever elusive balance.

## Author Contributions

AS is the sole author and wrote the first draft.

## Conflict of Interest Statement

The author declares that the research was conducted in the absence of any commercial or financial relationships that could be construed as a potential conflict of interest.
